# Generation of a global synthetic tropical cyclone hazard dataset using STORM

**DOI:** 10.1038/s41597-020-0381-2

**Published:** 2020-02-06

**Authors:** Nadia Bloemendaal, Ivan D. Haigh, Hans de Moel, Sanne Muis, Reindert J. Haarsma, Jeroen C. J. H. Aerts

**Affiliations:** 1 Institute for Environmental Studies (IVM), Vrije Universiteit Amsterdam, 1081 HV Amsterdam, The Netherlands; 2 School of Ocean and Earth Science, National Oceanography Centre, University of Southampton, European Way, Southampton, SO14 3ZH United Kingdom; 3 Deltares, 2600 MH Delft, The Netherlands; 4 Royal Netherlands Meteorological Institute (KNMI), 3731 GA De Bilt, The Netherlands

**Keywords:** Atmospheric dynamics, Natural hazards, Environmental impact

## Abstract

Over the past few decades, the world has seen substantial tropical cyclone (TC) damages, with the 2017 Hurricanes Harvey, Irma and Maria entering the top-5 costliest Atlantic hurricanes ever. Calculating TC risk at a global scale, however, has proven difficult given the limited temporal and spatial information on TCs across much of the global coastline. Here, we present a novel database on TC characteristics on a global scale using a newly developed synthetic resampling algorithm we call STORM (Synthetic Tropical cyclOne geneRation Model). STORM can be applied to any meteorological dataset to statistically resample and model TC tracks and intensities. We apply STORM to extracted TCs from 38 years of historical data from IBTrACS to statistically extend this dataset to 10,000 years of TC activity. We show that STORM preserves the TC statistics as found in the original dataset. The STORM dataset can be used for TC hazard assessments and risk modeling in TC-prone regions.

## Abstract (summary)

**Table:** 

Measurement(s)	cyclone
Technology Type(s)	computational modeling technique
Factor Type(s)	year • basin
Sample Characteristic - Environment	climate system
Sample Characteristic - Location	Earth (planet)

Machine-accessible metadata file describing the reported data: 10.6084/m9.figshare.11733585

## Background & Summary

Tropical cyclones (TCs), also referred to as hurricanes or typhoons, are one of the deadliest natural disasters, significantly impacting people, economies and the environment in coastal areas when they make landfall. The 2017 Atlantic Hurricane season became the costliest season ever with Hurricanes Harvey, Irma and Maria’s combined overall losses estimated around $220 billion^1^. It is therefore crucial to support risk mitigation efforts with reliable TC risk assessments. Performing such risk assessments can, however, be challenging. This is because TCs are relatively rare, with around 90 (±10) formations per year globally^2^, of which on average 16 TCs make landfall with wind speeds greater than 33 m/s^3^. Moreover, when making landfall, TCs generally affect a relatively small stretch of coastline (<500 km^4^), and impacts are higher in urbanized areas compared to rural or uninhabited regions of the world. In addition, reliable TC datasets are only available from 1980 onwards, meaning that for many coastal regions there may not even be a single landfalling TC event in available datasets. Correspondingly, many regions lack information about potential magnitudes and probabilities of TCs, particularly for extreme TCs (i.e. high return periods, low probabilities). This complicates reliable TC risk assessments and corresponding TC risk management.

One way to overcome both temporal and spatial data scarcity for low probability TCs is to extend the historical record. One such approach is by using paleo climate records from coastal sediments which can reveal periods of past coastal floods driven by TCs^5,6^. These records, however, are only available for small coastal stretches, and this method of collecting coastal sediment records is not feasible for upscaling at the global scale. Another method that has been widely explored in the past decades is the generation of synthetic TC tracks^7–9^. In such an approach, TC tracks and intensities are statistically resampled and modeled from an underlying dataset, which can be either historical TC tracks^8,10,11^ or meteorological datasets from climate models^12^. This creates a new TC, having similar characteristics as the ones in the underlying dataset. This sampling method can be repeated for a large number of years, hereby creating a larger TC dataset including TCs with high return periods.

There are two main synthetic model approaches, namely: (i) a coupled statistical-dynamical model; (ii) and a fully statistical model. Coupled statistical-dynamical models use autoregressive functions for certain parts of the simulation (e.g. only the track^8^), whilst the rest is simulated using a dynamical model. Examples of such models include the models developed by Emanuel, *et al*.^8^ and Lee, *et al*.^13^. Both models are run on the global scale, however they require a substantial number of input variables and are computationally intensive. Fully statistical models use Markov Chains^14^ for both the track generation and intensity simulation. These models generally require a limited number of input variables, and are less computationally intensive, making them easily applicable. This method has been used by for instance Haigh, *et al*.^10^, Vickery, *et al*.^7^, Hardy, *et al*.^15^ and James and Mason^16^, but only on local to regional scales.

In this paper, we present the synthetic algorithm Synthetic Tropical cyclOne geneRation Model (STORM), and apply it to develop a global dataset representative of 10,000 years of TC activity under present climate conditions. STORM applies a fully statistical approach based on a modified version of the methodology set out in James and Mason^16^ and Haigh, *et al*.^10^. STORM includes information on TC track, intensity, and size, which can be used to assess TC hazards such as wind, waves, and storm surge. Moreover, STORM requires a minimum number of input variables and is designed in a way that it can be applied to any meteorological dataset to statistically resample and model TC tracks and intensities. Here, we demonstrate STORM using best-track historical data from the International Best Track Archive for Climate Stewardship (IBTrACS)^17^. The length of the resulting dataset (i.e. 10,000 years under the same climate conditions) enables proper statistical analysis of return periods of various landfalling TCs. The dataset is particularly useful for TC risk assessments as it can serve as input for storm surge and wave impact modeling, and has characteristics important for wind damage assessments (maximum 10-meter wind speed).

## Methods

**Fig. 1:**
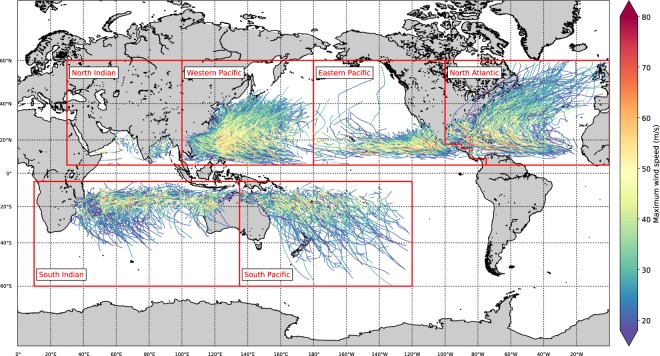
Overview of the different basins (red boxes; see also Table 1) and the tracks and intensities of the tropical cyclones in the IBTrACS dataset.

To create 10,000 years of synthetic TC data, there are three main stages, as follows:

1. **Data preparation and input:** Extract TCs from the source dataset IBTrACS (see Figs. 1 and 2, blue column) and this forms the input for the STORM model.

**Fig. 2:**
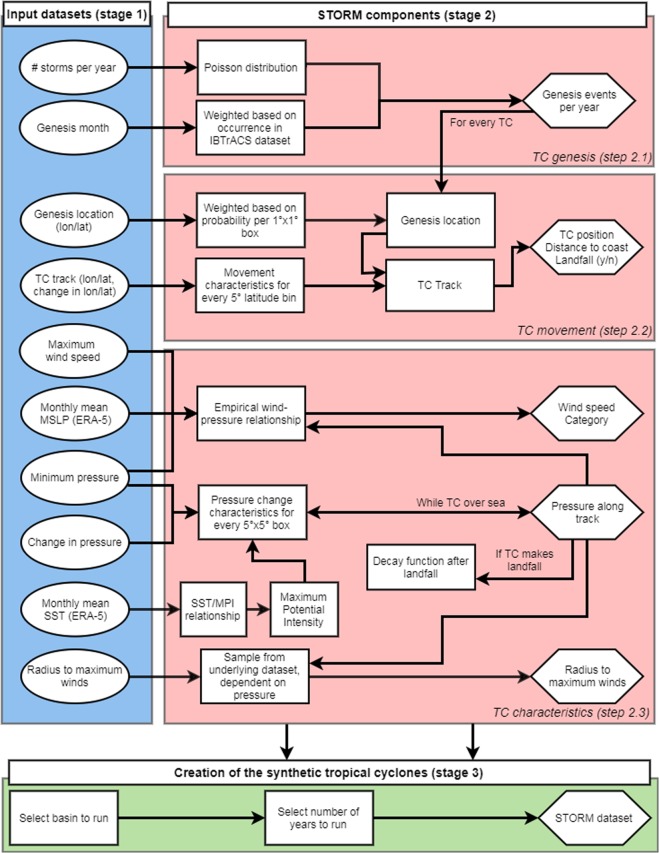
Flowchart with the extracted IBTrACS tropical cyclone (TC) characteristics (stage 1; in blue), the STORM components (stage 2; in red), and the creation of the synthetic tropical cyclones (stage 3; in green). Round boxes represent input data, square boxes represent the methodological steps taken to process this input data, and hexagonal boxes represent the output data.
2. **Fitting distributions and relationships:** The characteristics of the extracted storms are identified and pre-processed to create various distributions and relationships (see Fig. 2, red column); and
3. **Creating the synthetic TCs:** The distributions and relationships are used to create 10,000 years of TCs, with their corresponding characteristics (see Fig. 2, green column).

These three stages are explained in more detail below.

### Input datasets

The first stage is data preparation and input, which involves two steps. In step 1.1, as input data for STORM, we extract TCs from the global historical dataset IBTrACS^17^ for the time period 1980–2018 (38 years of data). We use data from 1980 onwards to comply with the modern era of satellite observations. IBTrACS is the unified dataset of the postseason best-track data produced by the tropical warning centers, for all the TC basins. Here, we use all basins except the South Atlantic. The basin domains are adapted from the basin domains used in the IBTrACS dataset (Table 1). The South Atlantic has been left out as there are too few TC formations in this basin for adequate distribution and relationships fitting. Prior to extracting the storms, we first unify the reported wind speeds to 10-minute average sustained wind speeds (U10; in m/s). This is done because the definition of these reported wind speeds differ per tropical warning center: 4 centers use either 1-minute or 3-minute averaging periods^17^. These wind speeds are multiplied by a factor of 0.88 to convert them to U10^18^. Next, for each basin, we extract the storms at all consecutive time steps where the U10 is greater than 18 m/s, or where the TC has not reached an extratropical cyclone-classification in the IBTrACS dataset. We selected the 18 m/s-threshold to comply with the tropical storm-classification on the Saffir-Simpson hurricane scale^19^. For convenience, we refer to this subset of storms as tropical cyclones (TCs) hereafter. We linearly interpolate all extracted data to 3-hourly values. The extracted tracks are shown in Fig. 1; an overview of all the extracted elements of the IBTrACS dataset is listed in Fig. 2 (blue column).

**Table 1:** Definition of the basin domains^17^ and tropical cyclone (TC) seasons^32^ used in this study. The basin domains are based on the basin domains used in the IBTrACS dataset.

Basin name	Abbreviation	Basin domain	TC season
Eastern Pacific	EP	5°–60°N 180°–coastline of North America on the North Atlantic	1 June–30 November
North Atlantic	NA	5°–60°N coastline of North America on the Eastern Pacific - 360°	1 June–30 November
North Indian	NI	5°–60°N 30°–100°E	1 April–30 June 1 September–30 November
South Indian	SI	5–60°S 10°–135°E	1 November–30 April
South Pacific	SP	5–60°S 135°–240°E	1 November–30 April
Western Pacific	WP	5–60°N 100°–180°E	1 May–30 November

The modeling of synthetic tracks also requires information on environmental conditions such as monthly mean MSLP and sea-surface temperatures (SST). Therefore, in stage 1, we extract MSLP and SST fields from the European Centre for Medium-Range Weather Forecasting (ECMWF)’s fifth generation climate reanalysis dataset ERA-5^20^. The spatial and temporal resolution of this dataset is 0.25° × 0.25° and 1-hourly. For both variables, we calculate the monthly mean values during the TC seasons, as defined in Table 1.

### STORM components

In the second stage, the extracted TC tracks and characteristics from IBTrACS along with the environmental conditions from ERA-5 are used as input to our synthetic resampling algorithm called Synthetic Tropical cyclOne geneRation Model (STORM). The STORM model follows three main steps that are visualized in Fig. 2 in the red column. In step 2.1, STORM samples the number of genesis events, and their corresponding genesis month, for every simulated year. In step 2.2, for each of these genesis events, a genesis location is determined, and, by adding consecutive changes in longitude and latitude, a synthetic track is formed. In step 2.3, TC characteristics such as minimum pressure, maximum wind speed, and radius to maximum winds are assigned along each of these tracks. These three steps are described in detail below.

### Tropical cyclone genesis

In step 2.1, we simulate the number of genesis events per year using a Poisson distribution, where the Poisson parameter λ is defined as the average number of TC formations a year in the input dataset. For the IBTrACS dataset, values of λ amount to 14.5 for the EP, 10.8 for the NA, 2.0 for the NI, 12.3 for the SI, 9.3 for the SP, and 22.5 for the WP. For each genesis event, we randomly assign it a genesis month, weighted by the genesis months per basin observed in the IBTrACS dataset.

### Tropical cyclone movement

In step 2.2, after determining the number of TC events in a year and assigning each a genesis month (see Section 2), we derive corresponding genesis locations for each TC event. This is based on weighted genesis locations per month from IBTrACS. For this, genesis locations are counted in 5° × 5° boxes and assigned to the box center point. Analogously, these points are interpolated (using cubic interpolation) to a 1° × 1° grid. The value these grid boxes is then used as weighting when sampling genesis locations. Lastly, the genesis location of the TC is sampled by selecting a random location (at 0.1° resolution) inside the 1° × 1° grid cell.

We then extract the changes (Δ) in the longitudinal (*ξ*) and latitudinal (*φ*) position of the TC eye at every time step t for every basin from IBTrACS. These Δ*ξ*_*t*_ and Δ*φ*_*t*_ are then grouped in 5° latitude sections per basin. For every bin, and using non-linear least squares, we fit a set of regression formulas following James and Mason^16^:

1a $$\Delta {\xi }_{t}={a}_{0}+{a}_{1}\Delta {\xi }_{t-1}$$

1b $${\xi }_{t}={\xi }_{t-1}+\Delta {\xi }_{t}+{\varepsilon }_{\xi },\,{\varepsilon }_{\xi } \sim N({\mu }_{{\varepsilon }_{\xi }},{\sigma }_{{\varepsilon }_{\xi }})$$

2a $$\Delta {\varphi }_{t}={b}_{0}+{b}_{1}\Delta {\varphi }_{t-1}+\frac{{b}_{2}}{{\varphi }_{t}}$$

2b $${\varphi }_{t}={\varphi }_{t-1}+\Delta {\varphi }_{t}+{\varepsilon }_{\varphi },\,{\varepsilon }_{\varphi } \sim N({\mu }_{{\varepsilon }_{\varphi }},\,{\sigma }_{{\varepsilon }_{\varphi }})$$

The residual term ε is drawn from a normal distribution of ε-values in IBTrACS. These ε-values are calculated as the difference between the fitted values of $\Delta {\xi }_{t}$ and $\Delta {\varphi }_{t}$ (Eqs. (1a) and (2a)) and the actual values of $\Delta {\xi }_{t}$ and $\Delta {\varphi }_{t}$ in IBTrACS.

### Tropical cyclone characteristics

In step 2.3, TC characteristics such as minimum pressure, maximum wind speed, and radius to maximum winds are assigned along each of the TC tracks. First, the conversion between the maximum U10 and minimum MSLP in a TC is modeled using the empirical wind-pressure relationship (WPR)^21,22^:

3 $${V}_{t}=a{\left({P}_{env}-{P}_{t}\right)}^{b}$$

where *V*_*t*_ and *P*_*t*_ are the maximum U10 and minimum MSLP at time step t, respectively. To estimate the variables *a* and *b* for every month in every basin, we extract maximum U10 and minimum MSLP from IBTrACS at every time step. The corresponding environmental pressure *P*_*env*_ is taken from the monthly mean MSLP fields from ERA-5. Equation (3) is then fitted to this data using non-linear least squares.

Second, we model the TC intensity along each synthetic track. A crucial feature in modeling this TC intensity is inhibiting TCs from growing too intense. In the STORM model, the TC intensity is constrained based on the maximum potential intensity (MPI)^23,24^, which is a measure of the theoretical maximum TC intensity possible at a location, dependent on environmental factors and atmospheric conditions. This implementation also captures the tendency of TCs to start weakening when they approach the MPI, as well as the tendency of TCs to weaken faster at higher latitudes^16^. The MPI is computed per month and per basin. First, we calculate the difference between the environmental pressure *P*_*env*_ and the TC’s central pressure P from the IBTrACS data. This value is also known as the TC pressure drop (${P}_{env}-P$, in hPa). Together with the pressure drop, we store the monthly mean SST (in 0.1 °C) corresponding to the location of the TC eye. Subsequently, we group these monthly mean SSTs in 0.1 °C bins together with their corresponding pressure drop values. We then fit the mean SST and maximum pressure drop per bin to Eq. (4) ^25^:

4 $${P}_{env}-P=A+B{e}^{C\left(SST-{T}_{0}\right)},\;\,{T}_{0}=30.{0}^{\circ }{\rm{C}}$$

The coefficients A, B and C are estimated using non-linear least-squares. Using this formula, we can calculate the maximum pressure drop at every 0.25° × 0.25° SST grid point. In the final step, we subtract this maximum pressure drop from the *P*_*env*_ fields to derive the MPI. To inhibit unrealistically low MPI values, the MPI values are bounded by the lowest MPI value per basin and per month derived by Bister and Emanuel^26^.

After calculating the MPI at every 0.25° × 0.25° grid point for every month, we model changes in P using an autoregressive formula similar to James and Mason^16^ (Eq. (5a)). For this, we first extract changes in central pressure at every time step (Δ*P*_*t*_) from IBTrACS and fit this to Eq. (5a). The values of the coefficients c_0_, c_1_ and c_2_ are deduced per month for every 5° × 5° box within a basin. The residual term *ε*_*p*_ is calculated as the difference between the fitted value of Δ*P*_*t*_ (Eq. (5a)) and the actual value of Δ*P*_*t*_ in IBTrACS. $\Delta {P}_{0.01}$ and $\Delta {P}_{0.99}$ represent the 1th- and 99th- percentile values of Δ*P*_*t*_, respectively.

5a $$\Delta {P}_{t}={c}_{0}+{c}_{1}\Delta {P}_{t-{\rm{1}}}+{c}_{2}{e}^{-{c}_{{\rm{3}}}X},\quad \,{c}_{2} > 0,\,X=\max \{0,\,{P}_{t}-MPI\}$$

5b $$\Delta {P}_{0.01}\le \Delta {P}_{t}+{{\rm{\varepsilon }}}_{p}\le \Delta {P}_{0.99},\quad {{\rm{\varepsilon }}}_{p} \sim N({\mu }_{{{\rm{\varepsilon }}}_{p}},{{\rm{\sigma }}}_{{{\rm{\varepsilon }}}_{p}})$$

5c $${P}_{t}={P}_{t-1}+\Delta {P}_{t}+{\varepsilon }_{p}$$

At genesis, we set U10 = 18 m/s, and calculate the corresponding genesis pressure *P*_0_ using the WPR (Eq. (3)). The first-step change in *P*, Δ*P*_0_, is drawn from a normal distribution fitted around the Δ*P*_0_ in IBTrACS. To inhibit the synthetic TC to dissipate directly after TC genesis, we force the synthetic TC to intensify (Δ*P*_0_ < 0) for the first 2 to 5 time steps. This amount of time steps is used to calibrate the average lifetime of a TC per basin.

A TC generally starts decaying after making landfall. In order to derive landfall (and distance to coast) for each time step of the TC in the STORM model, a land mask is created for each basin at 0.1° resolution using the Python 2.7 Basemap module. This module uses the GSHHS dataset^27^ for the coastline data^28^. When the TC eye is over land for at least three time steps (totaling 9 hours), the decay in TC wind speed in the STORM model is modelled following Kaplan and DeMaria^29^, who assume that the TC intensity decreases as a function of the time and distance the TC has covered whilst being over land:

6a $$V\left({t}_{L}\right)={V}_{b}+\left(R{V}_{0}-{V}_{b}\right){e}^{-\alpha t}-C,\;\,R=0.9,\,{V}_{b}=26.7\,kt,\,\alpha =0.095\,{h}^{-1}$$

6b $$C=m\left[{\rm{ln}}\left(\frac{D}{{D}_{0}}\right)\right]+b,\;\,D\gg 1,\,{D}_{0}=1$$

6c $$m=\widetilde{{c}_{1}}{t}_{L}({t}_{0,L}-{t}_{L}),\widetilde{{c}_{1}}=0.0109\,kt{h}^{-2},\,{t}_{0,L}=150\,h$$

6d $$b={d}_{1}{t}_{L}\left({t}_{0,L}-{t}_{L}\right),\,{d}_{1}=-\,0.0503\,kt{h}^{-2}$$

here, V is the maximum sustained wind speed (in kt) of the TC at any time step t_L_ after landfall. V_0_ is the wind speed at landfall. D represents the distance from the landfall location (in km). When the TC moves back over ocean or if the TC is over land for less than three time steps (9 hours), changes in intensity are modeled according to the set of Eq. (5).

**Fig. 3:**
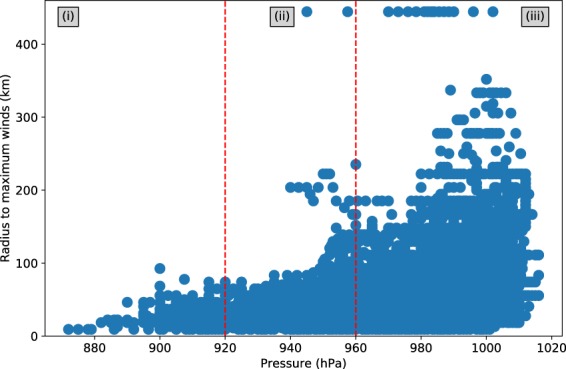
Scatterplot of the tropical cyclone’s minimum pressure (in hPa) against the radius to maximum winds (in km). The data are taken from the IBTrACS dataset. The red lines indicate the selected sub-sections.

Finally we derive values for Rmax. From IBTrACS we extract the Rmax (in km) and *P* for every time step whenever available, and group this together in one global dataset. This is done because for some basins, such dataset would be too small to adequately draw a new set of values from. In Fig. 3, we see that the relatively intense TCs tend to have a smaller Rmax, which is consistent with Shen^30^. For relatively weak TCs, we observe a much wider range of Rmax in IBTrACS. There is, however, no specific relationship between Rmax and P.

To calculate Rmax in STORM, we therefore split the dataset in three subsets:

(i) Rmax for P < 920 hPa (relatively small radii);
(ii) Rmax for 920 hPa < P < 960 hPa (transition), and
(iii) Rmax for P > 960 hPa (wide range of Rmax).

Sampling of Rmax at every time step can result in large sudden changes in Rmax. To avoid this, we sample values for Rmax at three distinct moments: at genesis (Rmax_gen_), at the moment of peak intensity (minimum P) (Rmax_peak_), and at dissipation (Rmax_dis_). Rmax values for the intermediate time steps is interpolated from these values (see the set of Eq. (7)). If Rmax_peak < _Rmax_gen_, this meets the observations that Rmax tends to decrease as intensity increases, and we let Rmax linearly decrease between Rmax_gen_ and Rmax_peak_ (Eq. (7a)). If Rmax_peak_ ≥ Rmax_gen_, we set Rmax_peak_ = Rmax_gen_, as otherwise the Rmax and TC intensity would both increase at the same time which is generally not the case^30^. In a similar manner, we let Rmax linearly increase if Rmax_dis_ > Rmax_peak_, and we set Rmax_dis_ = Rmax_peak._ if Rmax_dis_ ≤ Rmax_peak_. This way, the Rmax does not decrease while the TC is weakening, a property usually attributed to intensifying TCs^30^. This results in the following set of equations:

7a $$Rmax\left(t\right)=\frac{\left(Rma{x}_{peak}-Rma{x}_{gen}\right)\ast t}{{t}_{peak}}+Rma{x}_{gen}\quad \quad \quad t\le {t}_{int}$$

7b $$Rmax\left(t\right)=\frac{(Rma{x}_{dis}-Rma{x}_{peak})\ast (t-{t}_{dis})}{{t}_{dis}-{t}_{peak}}+Rma{x}_{dis}\quad \quad \quad t > {t}_{int}$$

### Creation of the synthetic tropical cyclones

**Fig. 4:**
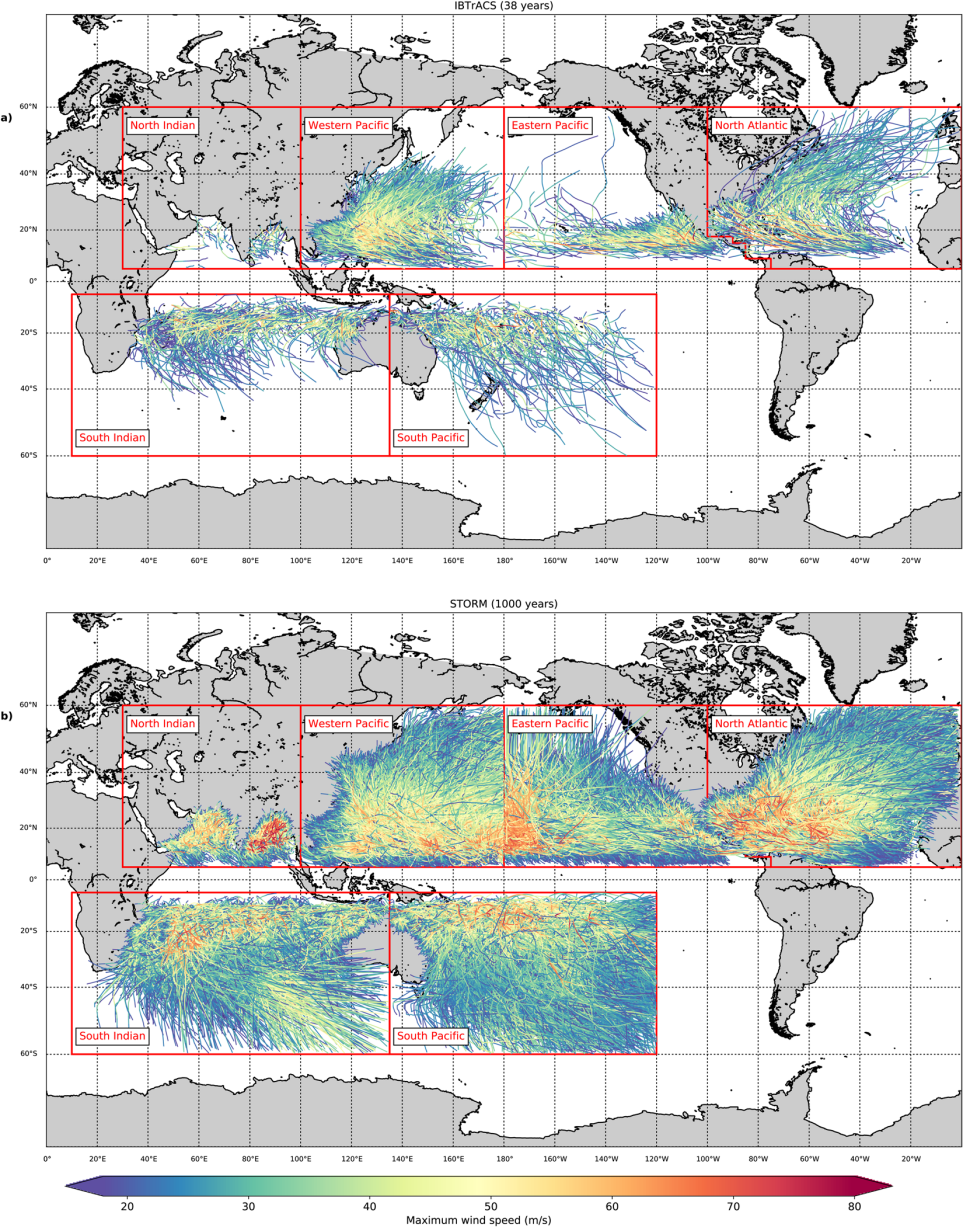
Overview of tropical cyclone tracks in IBTrACS and the STORM dataset. The top panel represents 38 years (1980–2018) of tracks in the IBTrACS dataset (**a**), the bottom panel represents a random period of 1,000 years of tropical cyclone tracks in the STORM dataset (**b**). Colors indicate the maximum wind speed of the tropical cyclone.

In the third stage (Fig. 2, green column), we create 10,000 years of synthetic TCs based on the present climate-conditions from the IBTrACS dataset. This is done by running the STORM model, consisting of a series of Python programs for each of the components described above, on the Lisa Computer Cluster (www.surf.nl). We split the 10,000 years in 10 separate runs of 1,000 years each, for each basin. Model runs of 1,000 years take on average a few hours to run, but specific run times depend on the selected basin. For time periods in the order of decades, it is also possible to run the STORM model on a regular desktop computer or laptop. Figure 4 shows the 38 years of TC activity in the input dataset IBTrACS (Fig. 4a) versus the synthetic TC tracks from such a random 1,000 year STORM model run (Fig. 4b). The STORM dataset provides a high global coverage of TCs compared to the original IBTrACS dataset, hereby ensuring that all coastal segments in TC-prone regions see multiple landfalling events in the STORM dataset. The STORM model outcomes are further discussed in Section 4.

## Data Records

The 10,000 year TC STORM dataset, based on the present climate, is publicly accessible and can be found on the 4TU.Centre for Research Data repository^31^ (10.4121/uuid:82c1dc0d-5485-43d8-901a-ce7f26cda35d). The dataset is split in separate files per basin, with each .txt-file containing 1,000 years of simulations (i.e. 10 files per basin). Each .txt-file consists of a series of arrays, with each array being a single time step (3-hourly) for a synthetic TC. The structure of the arrays is given in Table 2. Every entry in an array is comma-separated.

**Table 2:** Overview of entries in the dataset.

Entry	Variable name	Unit	Notes on variable
1	Year	—	Starts at 0
2	Month	—	
3	TC number	—	For every year; starts at 0.
4	Time step	3-hourly	For every TC; starts at 0.
5	Basin ID	—	0 = EP, 1 = NA, 2 = NI, 3 = SI, 4 = SP, 5 = WP
6	Latitude	Deg	Position of the eye.
7	Longitude	Deg	Position of the eye. Ranges from 0-360°, with prime meridian at Greenwich.
8	Minimum pressure	hPa	
9	Maximum wind speed	m/s	
10	Radius to maximum winds	km	
11	Category	—	On the Saffir-Simpson scale^19^
12	Landfall	—	0 = no landfall, 1 = landfall
13	Distance to land	km	

## Technical Validation

### Tropical cyclone characteristics

**Fig. 5:**
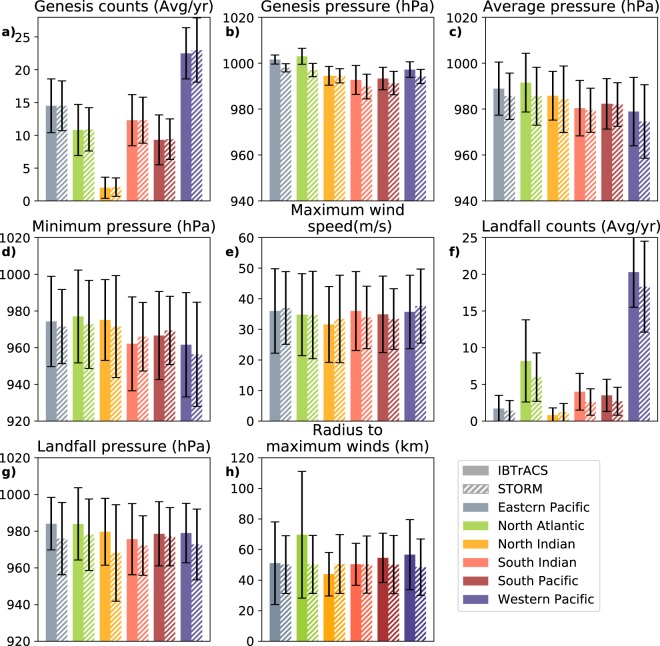
Bar charts showing the mean value of each of the different tropical cyclone characteristics, as listed in Table 3. Black lines represent the error bar, given as one standard deviation from the mean. Each of the colors represents a different basin. Solid bars represent IBTrACS data, dashed bars represent STORM data.

To assess the performance of the STORM algorithm, we compare the TC characteristics from the IBTrACS dataset (which serve as input for STORM) to those in STORM. The resulting statistics are listed in Table 3 and plotted in Fig. 5. Because the IBTrACS dataset serves as input for the generation of the STORM dataset, there exists a certain dependency between the two datasets. For this reason, we do not test for significant differences. Instead, we evaluate the performance of the STORM model by demonstrating that the mean values of various TC characteristics are within one standard deviation from those found in the IBTrACS dataset.

**Table 3:** Distributions of tropical cyclone characteristics in the IBTrACS and STORM datasets per basin. The time period 1980–2018 is used for the IBTrACS dataset (38 years of data). For the STORM dataset, we calculated the mean and standard deviation (between brackets) for a random selection of 1000 times 38 years of data. The n-value is given as the second number between brackets.

		Eastern Pacific	North Atlantic	North Indian	South Indian	South Pacific	Western Pacific	Global
Genesis events (Avg/yr)	IBTrACS	14.5 (4.1; 38)	10.8 (3.9; 38)	2.0 (1.6; 38)	12.3 (3.9; 38)	9.3 (3.8; 38)	22.5 (3.9; 38)	71.3 (8.4; 38)
	STORM	14.5 (3.8; 38000)	10.9 (3.3; 38000)	2.1 (1.4; 38000)	12.3 (3.5; 38000)	9.4 (3.1; 38000)	23.0 (4.9; 38000)	72.3 (8.6; 38000)
Genesis pressure (hPa)	IBTrACS	1001.6 (2.0; 418)	1003.0 (3.5; 410)	994.5 (4.1; 75)	992.7 (6.3; 449)	993.3 (4.9; 351)	997.2 (3.4; 853)	997.4 (5.6; 2556)
	STORM	998.0 (1.8; 551839)	997.0 (2.9; 413801)	994.5 (3.1; *76107)*	989.8 (5.4; 468103)	991.3 (5.1; 355663)	994.2 (3.1; 874377)	994.3 (4.7; 2739890)
Average pressure along track (hPa)	IBTrACS	988.9 (11.6; 424)	991.5 (12.8; 410)	985.8 (10.6; 75)	980.4 (12.1; 465)	982.3 (11.0; 352)	978.9 (14.9; 854)	983.5 (13.9; 2580)
	STORM	985.6 (10.1; 551839)	985.6 (12.6; 413801)	984.2 (14.5; 76107)	979.5 (9.6; 468103)	982.0 (9.5; 355663)	974.6 (16.0; 874377)	980.5 (13.5; 2739890)
Minimum pressure along track (hPa)	IBTrACS	974.3 (24.6; 424)	977.0 (25.3; 410)	975.1 (22.0; 75)	962.1 (25.6; 465)	966.6 (24.0; 352)	961.6 (28.4; 854)	967.3 (26.8; 2580)
	STORM	971.5 (20.2; 551839)	972.7 (24.0; 413801)	971.5 (27.8; 76107)	966.0 (18.7; 468103)	969.4 (18.6; 355663)	956.4 (28.4; 874377)	965.6 (24.4; 2739890)
Maximum wind speed along track (m/s)	IBTrACS	36.0 (13.8; 424)	34.8 (13.4; 410)	31.6 (12.4; 75)	36.0 (12.9; 465)	34.9 (12.5; 352)	35.7 (12.0; 854)	35.4 (12.8; 2580)
	STORM	37.0 (11.9; 551839)	34.7 (14.3, 413801)	33.4 (14.3; 76107)	33.9 (10.2; 468103)	33.4 (9.9; 355663)	37.6 (12.1; 874377)	35.7 (12.1; 2739890)
Total landfall counts (Avg/yr)	IBTrACS	1.7 (1.8; 38)	8.2 (5.6; 38)	0.8 (1.0; 38)	4.0 (2.5; 38)	3.5 (2.2; 38)	20.3 (4.8; 38)	38.6 (8.1; 38)
	STORM	1.4 (1.4; 38000)	6.0 (3.3; 38000)	1.2 (1.2; 38000)	2.6 (1.8; 38000)	2.7 (1.9; 38000)	18.3 (6.2; 38000)	32.2 (7.7; 38000)
Landfall pressure (hPa)	IBTrACS	984.1 (14.3; 64)	984.0 (19.7; 312)	979.7 (18.2; 32)	975.7 (19.4; 153)	978.6 (17.5; 134)	979.0 (16.2; 772)	979.9 (17.6; 1467)
	STORM	976.0 (19.7; 54247)	978.1 (19.5; 227302)	968.2 (26.3; 44041)	972.2 (16.2; 99244)	977.0 (15.9; 101171)	972.8 (19.3; 874377)	974.1 (19.3; 1221083)
Radius to maximum winds (km)	IBTrACS	51.1 (27.0; 218)	69.7 (41.4; 133)	43.9 (14.2; 36)	50.4 (13.7; 105)	54.6 (16.1; 49)	56.7 (22.9; 184)	55.7 (27.9; 725)
	STORM	50.2 (18.9; 551839)	50.3 (19.0; 413801)	50.6 (19.2; 76107)	50.2 (18.7; 468103)	50.4 (19.0; 355663)	48.5 (18.4; 874377)	49.7 (18.8; 2739890)

In general, there is good agreement between the two datasets. In most basins, the genesis pressure is lower in the STORM dataset compared to IBTrACS (Fig. 5b). This is likely due to the combination of setting the genesis wind speed at 18 m/s and the use of the wind-pressure relationship (see Section 2) to convert this wind speed to a pressure-equivalent. Despite these differences, we see that for all basins, the average pressure along the track in the STORM dataset closely corresponds to the average pressure in the IBTrACS dataset (Fig. 5c). More importantly, for the calculation of return periods of the peak intensity of a TC, we evaluate the minimum pressure and maximum wind speed along a track in both the STORM and IBTrACS datasets (Fig. 5d,e). We observe that, for all basins, these values correspond closely to those found in IBTrACS. The largest deviations in wind speed between the two datasets are found for the South Indian and the Western Pacific, with an underestimation of 2.1 m/s and an overestimation of 1.9 m/s in maximum wind speed, respectively. This demonstrates that the STORM model, embedded with the MPI constraint and the wind-pressure relationship (see Section 2), succeeds in reproducing the intensities found in IBTrACS. For the calculation of TC risk along the global coastline, it is important that STORM reflects the landfall counts per basin as well as the landfall pressure (Fig. 5f,g). In both datasets, we observe a relatively large standard deviation, indicating that there is a substantial year-to-year difference in landfall counts. This is also mirrored by STORM. However, on average, the average landfall counts of the two datasets fall within one standard deviation of each other. Considering Rmax, there is a small deviation between observed and modelled values (Fig. 5h). The largest deviations are seen for the North Atlantic basin, with an observed average Rmax of 69.7 km versus 50.3 km in the STORM model. This large deviation is likely caused by the sampling process used in the STORM model to calculate Rmax (see Section 2). All observed Rmax values were grouped in one global dataset, and from that dataset Rmax values were drawn corresponding to the TCs intensity. This grouping was done to overcome data scarcity in the smaller basins. Although procedure seems to work well for the other basins (i.e. small deviations from IBTrACS data), the larger Rmax values in the North Atlantic basin are diminished when grouping them together with the lower Rmax values in other basins. One way to overcome this would be to group and consecutively sample Rmax values per basin, however the Rmax dataset needs to be sufficiently large per basin.

Based on this comparison, we conclude that the STORM dataset performs sufficiently to be used for TC risk assessments and TC hazard analyses. Values of peak intensities and landfall pressures in the STORM dataset closely correspond to those found in the original IBTrACS data. The landfall counts also closely correspond to the ones in the IBTrACS dataset. However, there is a large year-to-year difference in annual landfall counts in both datasets, driving the large standard deviations found in both datasets.

### Spatial distribution of tropical cyclone tracks

**Fig. 6:**
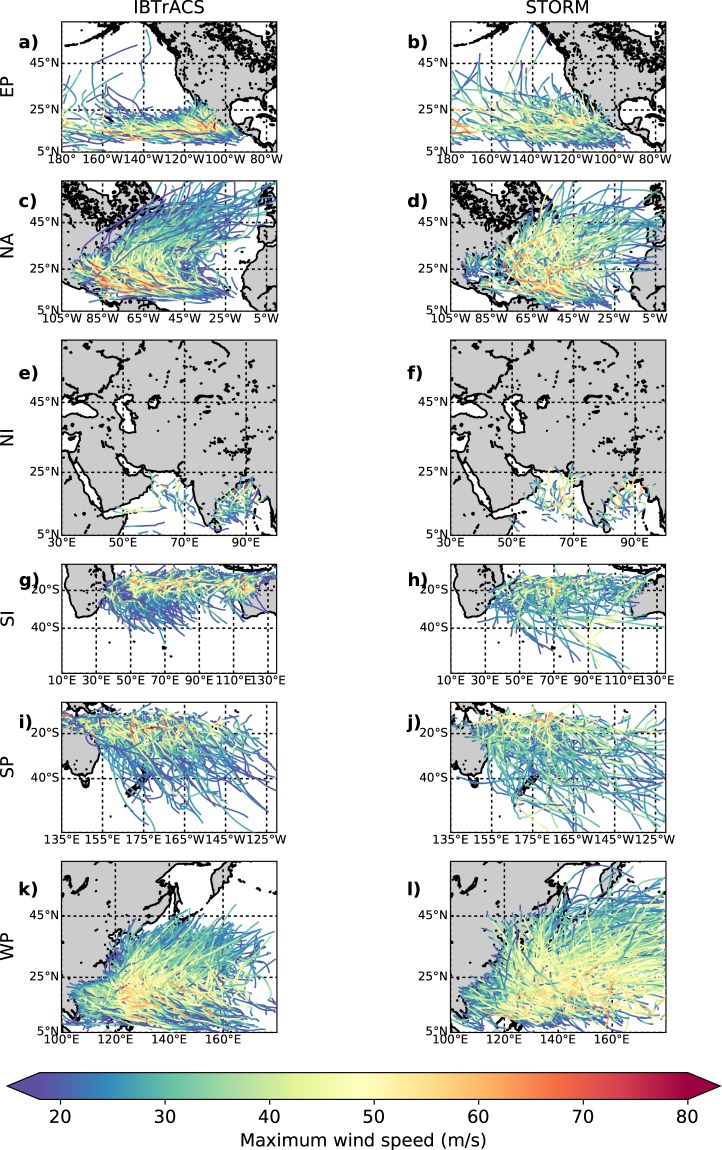
Overview of 38 years of tropical cyclone tracks in the IBTrACS and STORM dataset per basin. The left column represents 38 years (1980–2018) of tropical cyclone tracks in the IBTrACS dataset, the right column represents a random 38-year period in the STORM dataset. Colors indicate the maximum wind speed of the tropical cyclone.

Figure 6 shows the spatial distribution of the 38 years of TC tracks in the IBTrACS dataset (Fig. 6a) against a random selection of 38 years from the STORM dataset (Fig. 6b), per basin. In general, the location of peak intensity is captured well in the STORM dataset. These locations are more distinguishable when considering the 1,000 years of synthetic TC tracks in Fig. 4b. In Fig. 4b, we notice that these locations of peak intensity closely correspond with regions of highest SST per basin, such as the Caribbean Sea and the Gulf of Mexico in the North Atlantic or the Bay of Bengal in the North Indian. In the latter case, however, the intense maximum wind speeds are around 80 m/s in the 1,000-year dataset. These high wind speeds are the result of low MPIs in this basin, driven by high SSTs (see Section 2). These low MPIs, in turn, drive TC intensifications. The high wind speeds, however, do not point towards a significant overestimation of maximum wind speeds in this basin, as the average maximum wind speed along the track (see Table 3 and Fig. 5) closely corresponds to the ones found in the IBTrACS dataset.

In addition, Fig. 6 shows that the general patterns of TC tracks in the STORM dataset closely corresponds to those in the IBTrACS dataset. However, as the STORM model does not distinguish between tropical and extratropical systems, those longer-lived TCs in e.g. the South Indian (Fig. 6h) or the Western Pacific (Fig. 6l) are likely of extratropical nature once they reach higher latitudes, and should therefore be omitted from any TC-related analysis at such latitudes.

From Figs. 4 and 6, we can also observe the influence of basin boundary selection. We selected the basin boundaries such that they comply with the basin boundaries used in the IBTrACS dataset (see Section 2). The effect of the boundary selection is most prominent in the northern pacific basins (Western Pacific and Eastern Pacific): we observe that on the west side of the Eastern Pacific (between 180°W and 160°W; see Fig. 6b), TCs tend to grow more intense while moving westward. However they are cut off once they surpass 180°W. As there is little landmass in these regions, such TCs do not affect TC risk assessments, and as such we decided to leave the basin boundaries as is.

## Usage Notes

We have written the STORM algorithm in a modular and flexible way, so that it could be used to generate a large number of years of TC activity using any meteorological dataset as the input dataset. The resulting dataset with 10,000 years of TC activity can be used by anyone interested in researching TCs and TC risk over the open ocean and in coastal areas. Different aspects of TC hazards can be studied with this dataset, including wind and storm surge hazards. To this end, this dataset is applicable in various fields of study, including coastal modeling, flood risk assessments, and wind damage assessments. Because of its global coverage and the large number of TCs, there are also enough TCs to perform a risk assessment in regions rarely hit by TCs, such as small islands.

Here we have used IBTrACS and ERA-5 as input datasets for the STORM model, but one could also use, for example, high resolution global climate models. Such meteorological dataset should be a realistic representation of TC characteristics such as forward speed and direction, U10 and MSLP fields, and the radius to maximum winds. In addition, realistic monthly mean MSLP and SST fields are necessary for the modeling of environmental effects.

It is, however, important to note that the presented STORM dataset is based on the average climate conditions of the last 38 years and does not capture (multi)-decadal variability on longer time scales. In addition, the STORM model statistically resamples the same climate conditions as the input dataset. To this end, the STORM dataset as presented here cannot be used to assess climate change impacts over longer time scales. We recommend end-users interested in modeling synthetic TCs under future climate scenarios to either (i) re-run the STORM model with a future climate-dataset; or (ii) to use such dataset to estimate changes in TC characteristics under climate change compared to present climate, and to add this difference to a present climate-dataset such as IBTrACS (the delta method). We plan to do this in future work.

## References

[1] Munich Re. TOPICS GEO Natural Catastrophes 2017. 63 (2017).

[2] Emanuel K (2008). The Hurricane—Climate Connection. Bull. Amer. Meteor. Soc..

[3] Weinkle J, Maue R, Pielke R (2012). Historical Global Tropical Cyclone Landfalls. J. Climate.

[4] Pugh, D. & Woodworth, P. *Sea-Level Science: Understanding Tides, Surges, Tsunamis and Mean Sea-Level Changes*. (Cambridge University Press, 2014).

[5] Lin N, Lane P, Emanuel KA, Sullivan RM, Donnelly JP (2014). Heightened hurricane surge risk in northwest Florida revealed from climatological-hydrodynamic modeling and paleorecord reconstruction. J. Geophyis. Res Atmos..

[6] Nott J, Hayne M (2001). High frequency of super-cyclones along the Great Barrier Reef over the past 5,000 years. Nature.

[7] Vickery PJ, Skerlj PF, Twisdale LA (2000). Simulation of Hurricane Risk in the U.S. Using Empirical Track Model. J. Struct. Eng..

[8] Emanuel K, Ravela S, Vivant E, Risi C (2006). A Statistical Deterministic Approach to Hurricane Risk Assessment. Bull. Am. Meteor. Soc..

[9] Powell M (2005). State of Florida hurricane loss projection model: Atmospheric science component. J Wind Eng Ind Aerod.

[10] Haigh ID (2014). Estimating present day extreme water level exceedance probabilities around the coastline of Australia: tropical cyclone-induced storm surges. Clim Dynam.

[11] Casson E, Coles S (2000). Simulation and extremal analysis of hurricane events. J Royal Stat. Soc..

[12] Lin N, Emanuel K, Oppenheimer M, Vanmarcke E (2012). Physically based assessment of hurricane surge threat under climate change. Nat. Clim. Change.

[13] Lee C-Y, Tippett MK, Sobel AH, Camargo SJ (2018). An Environmentally Forced Tropical Cyclone Hazard Model. JAMES.

[14] Kolmogorov AN (1937). Markov chains with a countable number of possible states. Bull. Mosk. Gos. Univ. Math. Mekh.

[15] Hardy TA, McConochie JD, Mason LB (2003). Modeling Tropical Cyclone Wave Population of the Great Barrier Reef. J Waterw Port C Div.

[16] James MK, Mason LB (2005). Synthetic Tropical Cyclone Database. J Waterw Port C Div.

[17] Knapp KR, Kruk MC, Levinson DH, Diamond HJ, Neumann CJ (2010). The International Best Track Archive for Climate Stewardship (IBTrACS) Unifying Tropical Cyclone Data. Bull. Am. Meteor. Soc..

[18] Harper, B. A., Kepert, J. D. & Ginger, J. D. Guidelines for converting between various wind averaging periods in tropical cyclone conditions. (World Meteorological Organization, 2008).

[19] Simpson RH, Saffir H (1974). The hurricane disaster-potential scale. Weatherwise.

[20] Hersbach, H. *et al*. Global Reanalysis: goodbye ERA-Interim, hello ERA5. 17–24 (2019).

[21] Harper, B. *Tropical Cyclone Parameter Estimation in the Australian Region: Wind-Pressure Relationships and Related Issues for Engineering Planning and Design - A Discussion Paper*. (2002).

[22] Atkinson, G. D. & Holliday, C. R. Tropical Cyclone Minimum Sea Level Pressure/Maximum Sustained Wind Relationship for the Western North Pacific. *Mon. Wea. Rev*. **105**, 421–427, doi10.1175/1520-0493(1977)105<0421:tcmslp>2.0.co;2 (1977).

[23] Emanuel KA (1987). The dependence of hurricane intensity on climate. Nature.

[24] Holland, G. J. The Maximum Potential Intensity of Tropical Cyclones. *J. Atmos. Sci*. **54**, 2519–2541, doi:10.1175/1520-0469(1997)054<2519:tmpiot>2.0.co;2 (1997).

[25] DeMaria, M. & Kaplan, J. Sea Surface Temperature and the Maximum Intensity of Atlantic Tropical Cyclones. *J. Climate* **7**, 1324–1334, https://doi.org./10.1175/1520-0442(1994)007<1324:sstatm>2.0.co;2 (1994).

[26] Bister M, Emanuel KA (2002). Low frequency variability of tropical cyclone potential intensity 1. Interannual to interdecadal variability. Journal of Geophysical Research: Atmospheres.

[27] Wessel P, Smith WHF (1996). A global, self-consistent, hierarchical, high-resolution shoreline database. J Geophys Res Solid Earth.

[28] Whitaker, J. S. *Matplotlib basemap toolkit*, https://matplotlib.org/basemap/api/basemap_api.html (2011).

[29] Kaplan, J. & DeMaria, M. A Simple Empirical Model for Predicting the Decay of Tropical Cyclone Winds after Landfall. *J. Appl. Meteorol*. **34**, 2499–2512, 10.1175/1520-0450(1995)034<2499:ASEMFP>2.0.CO;2 (1995).

[30] Shen, W. Does the size of hurricane eye matter with its intensity? *Geophys. Res. Lett*. **33**, 10.1029/2006GL027313 (2006).

[31] Bloemendaal N (2019). 4TU.Centre for Research Data.

[32] World Meteorological Organization. *FAQs - Tropical Cyclones*, https://public.wmo.int/en/About-us/FAQs/faqs-tropical-cyclones (2018).

